# Anomalous scaling of flexural phonon damping in nanoresonators with confined fluid

**DOI:** 10.1038/s41378-018-0041-2

**Published:** 2019-01-14

**Authors:** Subhadeep De, Narayana R. Aluru

**Affiliations:** 1 Department of Mechanical Science and Engineering, University of Illinois at Urbana-Champaign, Urbana, IL 61801 USA; 2 Beckman Institute for Advanced Science and Technology, University of Illinois at Urbana-Champaign, Urbana, IL 61801 USA

**Keywords:** NEMS, Carbon nanotubes and fullerenes, Nanofluidics

## Abstract

Various one and two-dimensional (1D and 2D) nanomaterials and their combinations are emerging as next-generation sensors because of their unique opto-electro-mechanical properties accompanied by large surface-to-volume ratio and high quality factor. Though numerous studies have demonstrated an unparalleled sensitivity of these materials as resonant nanomechanical sensors under vacuum isolation, an assessment of their performance in the presence of an interacting medium like fluid environment is scarce. Here, we report the mechanical damping behavior of a 1D single-walled carbon nanotube (SWCNT) resonator operating in the fundamental flexural mode and interacting with a fluid environment, where the fluid is placed either inside or outside of the SWCNT. A scaling study of dissipation shows an anomalous behavior in case of interior fluid where the dissipation is found to be extremely low and scaling inversely with the fluid density. Analyzing the sources of dissipation reveals that (i) the phonon dissipation remains unaltered with fluid density and (ii) the anomalous dissipation scaling in the fluid interior case is solely a characteristic of the fluid response under confinement. Using linear response theory, we construct a fluid damping kernel which characterizes the hydrodynamic force response due to the resonant motion. The damping kernel-based analysis shows that the unexpected behavior stems from time dependence of the hydrodynamic response under nanoconfinement. Our systematic dissipation analysis helps us to infer the origin of the intrinsic dissipation. We also emphasize on the difference in dissipative response of the fluid under nanoconfinement when compared to a fluid exterior case. Our finding highlights a unique feature of confined fluid–structure interaction and evaluates its effect on the performance of high-frequency nanoresonators.

## Nanoresonator: wet nanotubes

Understanding the response of carbon nanotube-based nanomechanical resonators when in contact with a fluid could broaden their applications range. Low-dimensional materials are attractive for nanoscale sensing because they have a very high resonant frequency, enabling high quality factors. Carbon nanotubes are one such material, but their resonant behavior has rarely been studied when in contact with a fluid. Now, Subhadeep De and Narayana Aluru from University of Illinois at Urbana-Champaign study the nanomechanical resonance of single-walled carbon nanotubes under two conditions: with fluid passing through, and with fluid surrounding. They analyze dissipation losses in these systems, with a view to widening the applications range of carbon nanotube resonators used in chemical and biological environments.

## Introduction

In the pursuit of miniaturization of devices, low-dimensional nanomaterials like carbon nanotubes^1,2^, graphenes^3^, and monolayer transition metal dichalcogenides^4^ (TMDCs) have been drawing enormous attention for nearly two decades. One motivation towards miniaturization is making high-precision resonant mechanical sensors which can detect extremely small foreign mass, charge, force, etc.^5,6^. The unique optical^7,8^ and electronic properties^9–11^ of these low-dimensional materials can be coupled with their mechanical degrees of freedom to make the next generation sensors. During any such sensing process, ultra-high sensitivity can be attained when the resonator has low mass, large surface area, and very high resonant frequency^12,13^, all of which are inherently offered by 1D and 2D materials of nanometer dimension. However, different dissipative processes can interfere with the mechanical response of the resonators and limit their performance. The dissipation takes place due to the coupling of the resonant motion of interest, either with other internal degrees of freedom in the resonator, classified as an intrinsic source or with the external environment, classified as an extrinsic source. Typically, the ultimate performance of a resonator is evaluated under near-vacuum condition^13–15^, in which case only the intrinsic dissipation mechanisms are operative. But, such characterization may prove insufficient to assess their quality for applications involving biological^16,17^ and chemical^18–20^ sensing where an external gas or liquid environment is inevitably present. In such scenarios of a resonator in a fluid environment, estimating the net energy loss is not straightforward. Firstly, because the sources of dissipation can couple with each other and the coupling effect can manifest in each dissipation mechanism. Secondly, the response of the interacting media, fluid, in the parameter space of the resonant motion, which are gigahertz frequency and nanometer dimension is poorly understood and consequently, the resulting extrinsic dissipation, i.e., fluid dissipation, is difficult to estimate. In this regard, 1D or 2D high-frequency nanoresonators in a fluid environment is a platform not only to understand the individual dissipative processes and their coupling but also to probe the nature of fluid–structure interaction at the lower extremes of length and time scale.

The dominant mechanism behind dissipation varies significantly with the critical length of the resonator, the time scale of operation, and the mode of actuation. The intrinsic dissipation, for instance, in the case of beam resonators with micrometer cross-section and megahertz resonant frequency is dominated by thermoelastic damping^21,22^ and mediated by its phonons or thermal degrees of freedom. For smaller resonators of nanometer lengths operating at gigahertz frequencies, phonon-mediated dissipation due to Akhiezer mechanism^23,24^, anharmonic phonon scattering^25,26^, becomes more pronounced. For flexural vibration of 1D and 2D microresonators, nonlinear mode coupling, mode hybridization, and coupling to phonon bath are the dominant dissipation mechanisms^27–29^. Other forms of intrinsic dissipation could be mediated by the surface^30,31^ and structural defects^32^ and by the two-level states^33–35^ at low temperatures. Of all, phonon-mediated dissipation mechanisms are of major concern because it is ubiquitous to any resonator at room temperature. Viscous damping starts playing a significant role when the resonator is coupled with the fluid environment. The fluid can be placed, either outside the volume of the resonator or encapsulated in a channel inside the resonator. In the case of exterior fluid, the fluid damping is generally orders of magnitude higher than intrinsic damping and becomes a more important concern. This motivated the characterization of fluid damping interacting with vibrating micro-nano structures^36^ and nanoparticles^37^. It has been established that even “viscous” fluid can exhibit “viscoelastic” nature if the time scale for measurement becomes comparable to molecular timescales in the fluid. To account for the frequency dependent fluid response, a fluid relaxation parameter is incorporated in the constitutive relationship between shear stress and strain rate, and the classical Navier–Stokes (NS) equations are modified accordingly^38,39^. The effect of the fluid relaxation was observed in the case of gases through vibrating micro-nano resonators at kilohertz to megahertz frequencies^40^ and in case of liquids through vibrating nanoparticles at gigahertz frequencies^41^, respectively. The difference in the frequency range of observation is due to the difference in molecular timescales between gases and liquids. In all the cases of exterior fluid, the damping was consistently found to be directly proportional to the density or viscosity of the fluid. The case of fluid encapsulation exhibited some distinctive features like reduced fluid damping^42,43^ and nonmonotonic scaling with the fluid density or viscosity^44^. Those features, however, were shown to stem from the dynamics of the fluid under confinement. Because of the lower frequency in kilohertz and larger dimension of the fluid channel in micrometer, the oscillatory flow dynamics could be explained using classical NS equations without the fluid relaxation parameter. In this regard, the flexural phonon damping of nanotube resonators with confined fluid combines interesting and unexplored features of both phonon-mediated intrinsic damping and fluid damping. The confined fluid damping being low and comparable to the intrinsic dissipation, both become equally important. Each dissipation channel can exhibit some effect due to the presence of other entity. A large surface-to-volume ratio of these low-dimensional structures provides a scope to capture the effect. The flexural phonons interacting with the fluid can attain gigahertz frequencies, a range where the fluid response behavior is unknown. Additionally, the fluid interaction at the nanoscale structural interface bears the effect of slip^45,46^, density inhomogeneity^47^, and reorganization due to confinement^48^, all of which can effect the dissipation.

**Fig. 1:**
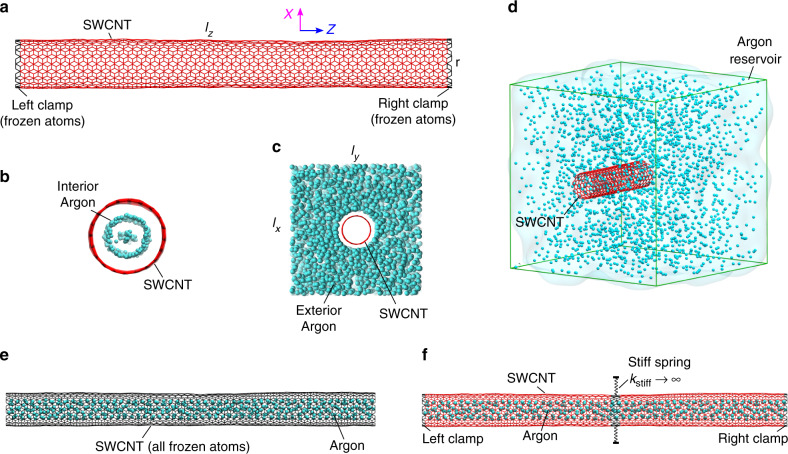
Snapshots of the simulation setups. **a** The side view of a single-walled carbon nanotube (SWCNT) with its axis aligned along the *Z* direction. The clamped atoms, which are kept frozen throughout the simulations, are colored in black. The rest of the atoms, colored in red, constitutes the vibrating part of the structure with allowed thermal motion. The side view of **b** an SWCNT with interior argon and **c** an SWCNT with exterior argon. The SWCNT is colored in red and the argon is colored in cyan. **d** An SWCNT in an argon reservoir. The edges of the cuboidal simulation box, which is periodic in all three directions, are colored in green. **e** An argon filled SWCNT with all of its atoms frozen. The frozen atoms are colored in black. **f** An SWCNT with interior argon and mid-section supported by stiff spring. The SWCNT atoms with thermal motion allowed and frozen are colored in red and black, respectively

We consider a 1D single-walled carbon nanotube (SWCNT) interacting with an argon environment at room temperature and study the damping of the fundamental flexural phonon with gigahertz resonant frequency. We set-up two different arrangements of the resonator–fluid system; in one, the fluid is solely confined inside the SWCNT (shown in Fig. 1b), and in other, fluid is placed outside the SWCNT (shown in Fig. 1c). Ringdown simulations of the SWCNT are carried out to compute the net dissipation during the resonant motion. The net dissipation, which is contributed by phonons and fluids in the system, is studied at different fluid densities for both the cases. A comparison of the fluid interior case with an empty SWCNT in vacuum revealed that dissipation due to phonons and fluid are comparable and that each of them and their cross-interaction can be of significance. First, we look at intrinsic dissipation due to phonons. We use the intrinsic dissipation computed for the empty SWCNT in vacuum to investigate if the associated dissipation mechanism is affected in the presence of interfacial fluid interaction. Next, we compute the fluid dissipation which results from the oscillatory flow generated in the fluid during the ringdown motion. We formulate the fluid dissipation in terms of a damping kernel which can be computed from the equilibrium fluctuations of the hydrodynamic force on the resonator. The fluid damping kernel is used to demonstrate (i) the effect of thermal motion of the resonator atoms at the interface (surface phonons) on the fluid dissipation and (ii) the time dependence of the hydrodynamic force from the fluid with nanoconfinement effects. The damping kernel is used to quantify the fluid dissipation and explain its unexpected scaling with the fluid density. Enabled by a systematic dissipation analysis, we discuss the origin of intrinsic dissipation. Moreover, we highlight the difference between the dissipative response of the nanoconfined fluid and the exterior fluid.

## Results

### System parameters

By varying reservoir pressure, argon can be confined inside the SWCNT with reduced densities ranging from ~0.01 to 0.57 (16.87–961.87 kg/m^3^). For the pressure range considered (~10–10^4^ bars), argon in the reservoir exists either in the vapor or the supercritical state^49,50^. For any higher pressure, argon turns solid and will prevent the filling of the SWCNT. To compare the dissipation in the interior and the exterior cases, a correspondence is established between the reservoir or bulk density $\left( {\rho _b^ \ast } \right)$ and the confinement density $\left( {\rho _i^ \ast } \right)$ of argon. The procedure is discussed in Supplementary Section 1.

**Fig. 2:**
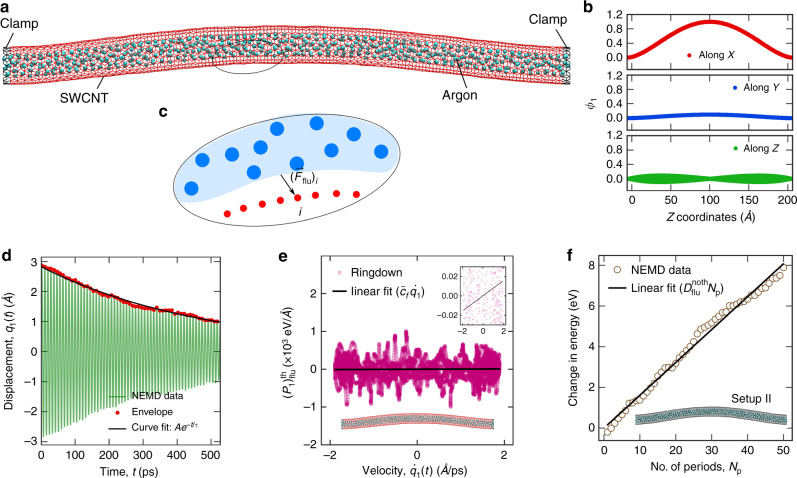
Dissipation calculations. **a** The side view of the deformed SWCNT with interior argon during the ringdown process. The SWCNT, the inside argon, and the clamped atoms are colored in red, cyan, and black, respectively. **b** The components of the computed mode shape *ϕ*_1_ of the fundamental flexural mode along the X, Y, and Z directions. **c** A schematic of the interaction between the resonator atoms (shown with red circles) and the fluid (shown in light blue). The black arrow indicates the net force $\left( {\vec F_{{\bf{flu}}}} \right)_i$ on the *i*th resonator atom from the fluid. **d** The decay of displacement amplitude of the fundamental flexural phonon with time during the ringdown process. The oscillatory green line is the displacement data, *q*_1_(*t*) of the fluid-coupled SWCNT obtained from nonequilibrium MD simulation. An envelope formed from the peaks of the displacement data is represented by the red circles. The envelope is fitted with an exponentially decaying curve, *Ae*^−*t*/*τ*^ shown with the black line, to characterize the decay time *τ*. **e** The net hydrodynamic force on the fundamental flexural mode of the resonator, $\left( {P_1} \right)_{{\mathrm{flu}}}^{th}$ plotted against its velocity $\dot q_1(t)$. The magenta circles are the data points obtained from nonequilibrium MD during the ringdown process. The black line is a linear fit to the data. The non-zero slope of the fitted line is visible from the inset. **f** The change in energy of the fluid-coupled resonator system averaged over a period of oscillation plotted against the number of periods, *N*_*p*_ in the case where all resonators atoms are frozen (setup II)

**Fig. 3:**
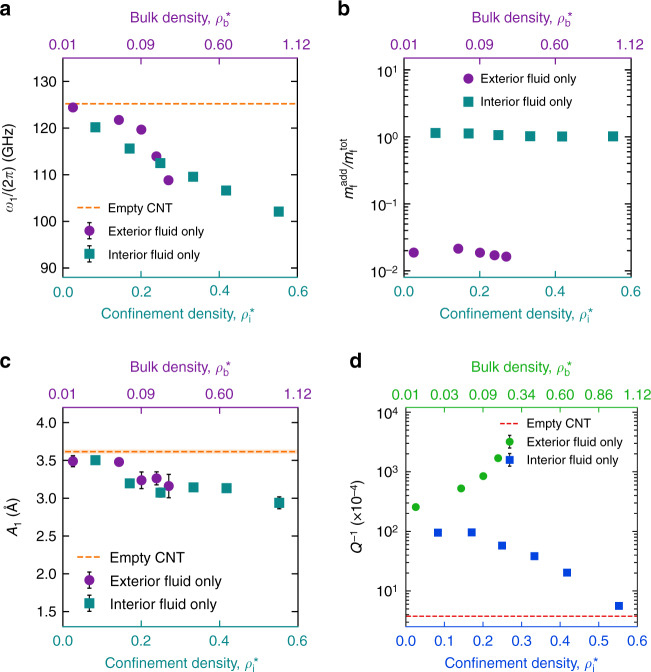
Fundamental flexural resonance characteristics  of the SWCNT. **a** The resonant frequency *ω*_1_ versus the fluid density ρ^*^. **b** The ratio of the added mass to the total mass of the fluid, $m_f^{add}{\mathrm{/}}m_f^{tot}$ versus the fluid density ρ^*^. **c** The initial amplitude *A*_1_ versus the fluid density ρ^*^. **d** The scaling of the inverse quality factor *Q*^−1^ with the argon density ρ^*^. For argon, *ρ* = ρ^*^ × 1687.5 kg/m^3^. In each case, the squares and the circles correspond to the interior and exterior argon case, respectively. The red dashed line corresponds to the empty SWCNT case with the spread in the data denoted by the thickness of the lighter solid red line. All the data points with error bars are obtained from ringdown simulation using nonequilibrium molecular dynamics

Ringdown is carried out for fluid densities ranging from $\rho _i^ \ast \sim 0.08$ to 0.55 (135.0–928.13 kg/m^3^) in the case of interior fluid, and $\rho _b^ \ast \sim 0.01$ to 0.19 (16.87–320.62 kg/m^3^) in the case of exterior fluid. A snapshot of the deformed SWCNT during the ringdown process is shown in Fig. 2a. The fundamental flexural phonon during the ringdown process is modeled as a damped harmonic oscillator. The parameters associated with the model are effective mass, effective stiffness (resonant frequency), and a damping constant. These parameters for the SWCNT in fluid can be related to the SWCNT in vacuum case as^51^

1 $$\omega _1 = \sqrt {\frac{{m^0}}{{m^0 + {\mathrm{\Delta }}m}}\left( {\omega _1^0} \right)^2 - \frac{{\nu _1^2}}{4}}$$

where *ω*_1_ and *ν*_1_ are the resonant frequency and the damping coefficient in fluid, $\omega _1^0$ is the resonant frequency in vacuum, *m*^0^ is the resonator mass, and Δ*m* is the added mass due to the fluid during the fundamental flexural motion. The derivation is shown in Supplementary Section 4c. A plot of the resonant frequency *ω*_1_ for different cases of argon coupled CNT system is shown in Fig. 3a. For the cases with interior argon, $Q^{ - 1} \ll 1$ and thus $\omega _1 \gg \nu _1{\mathrm{/}}2$. Therefore, the resonant frequency change with density in Fig. 3a is solely due to the added mass of the fluid. In the argon exterior cases, though the change in *ω*_1_ is majorly due to the added fluid mass, *ν*_1_ becomes significant and can contribute to the change for the highest densities.

Inserting the values of *m*^0^, $\omega _1^0$ and *ν*_1_ in Eq. (1), Δ*m*_1_ can be calculated. A plot of the effective mass of the fluid added $\left( {m_f^{add} = {\mathrm{\Delta }}m_1} \right)$ to the structure during the resonant motion is shown in Fig. 3b, in terms of $m_f^{add}{\mathrm{/}}m_f^{tot}$, where $m_f^{tot}$ is the total mass of fluid present in the fluid-coupled resonator system. The ratio $m_f^{add}{\mathrm{/}}m_f^{tot}$ in the interior case is found to be ~1.0, which is expected as the whole mass of fluid moves with the structure. In the exterior case, $m_f^{add}{\mathrm{/}}m_f^{tot}$ corresponds to the fraction of fluid mass that forms layers around the structure and moves with it.

The magnitude of the initial amplitude *A*_1_ depends on the extra energy imparted in the beginning as the part of the ringdown simulation. Figure 3c shows *A*_1_ at different densities for both interior and exterior argon cases. The amplitude values are close to each other because the initially imparted energy is kept approximately constant for all the cases. The small variation for the different cases is due to differences in the amount of damping and the fluid mass loading of the resonator.

The dissipation scaling for the interior and the exterior case is shown in Fig. 3d. The resonant frequency of the flexural phonon for all the cases is greater than ~100 GHz. To the best of our knowledge, no previous studies have reported dissipation in a fluid-coupled resonator system at such a high frequency. The dissipation in the interior cases is found to be lower, in some cases by almost 50 times, than the corresponding exterior cases. Also, in the exterior case, the total dissipation is found to increase with the increase in density. Both of these observations are qualitatively consistent with experiments on microresonators at KHz to MHz frequencies^36,44^. However, in the interior case, the total dissipation decreases with the increase in density. Conventionally, the increase in density of a bulk fluid is marked by increase in viscosity^36^ in the hydrodynamic limit^52^. Thus, increase in total dissipation in the exterior case can be explained by the increase in viscous force by the fluid on the SWCNT. By this argument, the scaling of total dissipation in the interior case is rather counter-intuitive. To elucidate the unexpected dissipation scaling, we carefully examine the individual sources contributing to the dissipation during the ringdown process.

### Sources of dissipation

During the resonant motion, the fundamental flexural phonon (*k* = 1) interacts with other phonons (*k* = 2, …, 3*N*) of the SWCNT and the fluid atoms. For the fluid-coupled resonator system (**sys**), the total dissipation, (*Q*^−1^)_**sys**_ (≡*Q*^−1^) is accounted by the intrinsic (**int**) dissipation governed by the phonons and extrinsic dissipation due to the fluid (**flu**). In the case of an empty SWCNT operating in vacuum (*v*), net dissipation, $\left( {Q^{ - 1}} \right)_{{\bf{int}}}^v$ during the fundamental flexural resonance is intrinsic and is solely mediated by phonons^25,26^. However, the presence of fluid (*f*) in the fluid-coupled resonator can change the nature of the interaction of the fundamental flexural phonon (*k* = 1) with other phonons at the fluid–structure interface. If that is true, the intrinsic dissipation in the fluid-coupled resonator can be different from $\left( {Q^{ - 1}} \right)_{{\bf{int}}}^v$ and is denoted by $\left( {Q^{ - 1}} \right)_{{\bf{int}}}^f$. Similarly, the extrinsic dissipation due to the fluid during the motion of the fundamental flexural phonon of the SWCNT can incorporate the effect of other phonons^53^ (*k* = 2, …, 3*N*) or thermal motion^54^ (*th*) of the atoms in the SWCNT. We denote the fluid dissipation by $\left( {Q^{ - 1}} \right)_{{\bf{flu}}}^{th}$ and $\left( {Q^{ - 1}} \right)_{{\bf{flu}}}^{noth}$ corresponding to the cases when other phonons (or atomic thermal motions) are present and absent in the SWCNT, respectively. With these definitions, the total dissipation in the fluid-coupled resonator system can be written as $\left( {Q^{ - 1}} \right)_{{\bf{sys}}} \equiv \left( {Q^{ - 1}} \right)_{{\bf{int}}}^f + \left( {Q^{ - 1}} \right)_{{\bf{flu}}}^{th}$.

During experiments or simulations on ringdown, the dissipation measured from the amplitude decay of the structure is (*Q*^−1^)_**sys**_. In our case of SWCNT resonator in fluid, (*Q*^−1^)_**sys**_ is obtained using nonequilibrium MD. The main challenge is the computation of the individual contributions namely $\left( {Q^{ - 1}} \right)_{{\bf{int}}}^f$ and $\left( {Q^{ - 1}} \right)_{{\bf{flu}}}^{th}$. For computing $\left( {Q^{ - 1}} \right)_{{\bf{flu}}}^{th}$, we use the total hydrodynamic force^55,56^ by the fluid on the fundamental flexural mode (*k* = 1) obtained from nonequilibrium MD during the ringdown. The modal hydrodynamic force, $\left( {P_1} \right)_{{\bf{flu}}}^{th}$ is calculated by projecting the hydrodynamic force, (*F*_*i*,*α*_)_**flu**_ on each resonator atom (shown in Fig. 2c) along the mode shape *ϕ*_1_. The slope of the hydrodynamic force with the velocity gives a damping coefficient $\tilde{c}_f\left( {\omega _1} \right)$ as shown in Fig. 2e. This damping coefficient can be seen as the real Fourier transform of a damping kernel *c*_*f*_(*t*) from time to frequency domain and evaluated at *ω*_1_. The formulation and subsequent derivation of the quality factor is shown in Supplementary Section 5. Using the damping coefficient, the inverse quality factor can be calculated as

2 $$\left( {Q^{ - 1}} \right)_{{\bf{flu}}}^{th} = \frac{{\omega _1\tilde c_f\left( {\omega _1} \right)}}{{\xi _1m^0\left( {\omega _1^0} \right)^2}}$$

**Fig. 4:**
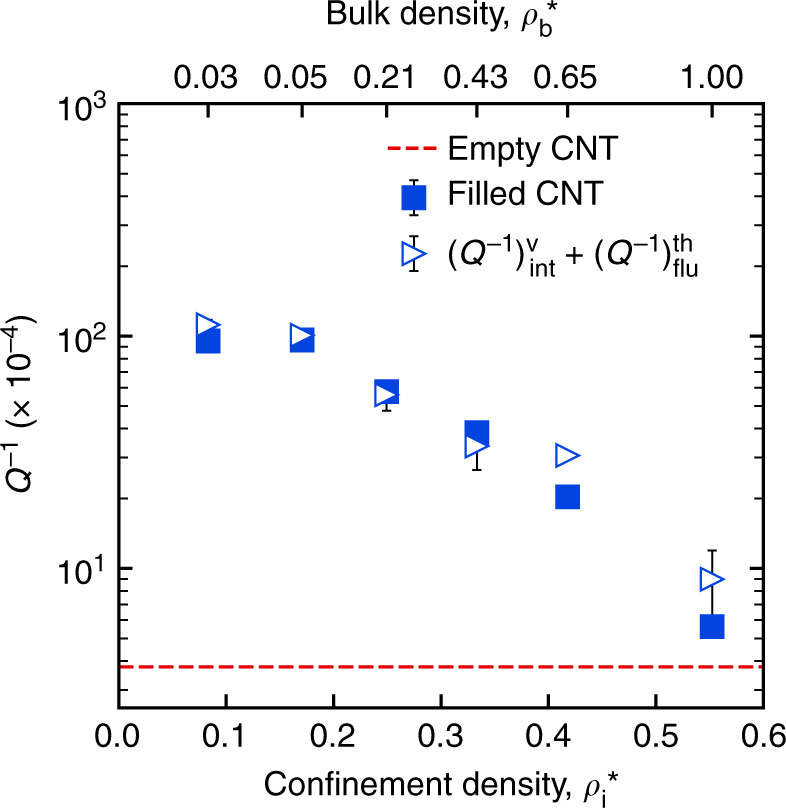
A comparison of the net dissipation *Q*^−1^ (filled blue squares) of SWCNT with interior argon with the sum $\left( {Q^{ - 1}} \right)_{{\bf{int}}}^v + \left( {Q^{ - 1}} \right)_{{\bf{flu}}}^{th}$ (empty triangles). Here $\left( {Q^{ - 1}} \right)_{{\bf{flu}}}^{th}$ is obtained from the same simulation and $\left( {Q^{ - 1}} \right)_{{\bf{int}}}^v$ is obtained from a separate simulation of empty SWCNT in vacuum. For argon, *ρ* = ρ^*^ × 1687.5 kg/m^3^. The red dashed line corresponds to dissipation in the empty SWCNT case. The data points with error bars are obtained from ringdown simulation using nonequilibrium molecular dynamics

Here, *ξ*_1_ is a fraction that depends on the mode shape. $\tilde c_f\left( {\omega _1} \right)$ is calculated at each fluid density during the ringdown process, which is then used to compute $\left( {Q^{ - 1}} \right)_{{\bf{flu}}}^{th}$ using Eq. (2). Now, there is no straightforward way to compute $\left( {Q^{ - 1}} \right)_{{\bf{int}}}^f$. So, as a first guess, we approximate $\left( {Q^{ - 1}} \right)_{{\bf{int}}}^f$ with $\left( {Q^{ - 1}} \right)_{{\bf{int}}}^v$ which is obtained by simulating ringdown of the empty SWCNT in vacuum. From $\left( {Q^{ - 1}} \right)_{{\bf{flu}}}^{th}$ and $\left( {Q^{ - 1}} \right)_{{\bf{int}}}^f$ approximated as $\left( {Q^{ - 1}} \right)_{{\bf{int}}}^v$, (*Q*^−1^)_**sys**_ $\left( { \approx \left( {Q^{ - 1}} \right)_{{\bf{int}}}^v + \left( {Q^{ - 1}} \right)_{{\bf{flu}}}^{th}} \right)$ is calculated. A comparison of the estimated (*Q*^−1^)_**sys**_ with *Q*^−1^ obtained directly from nonequilibrium MD simulation is shown in Fig. 4. A good agreement between them reveals that, $\left( {Q^{ - 1}} \right)_{{\bf{int}}}^f \approx \left( {Q^{ - 1}} \right)_{{\bf{int}}}^v$, is in fact true. This means that the energy exchange between the fundamental flexural phonon (*k* = 1) with the rest of the phonons (*k* = 2, …, 3*N*) is not influenced by the presence of the fluid at the fluid–structure interface. Also, the inverse scaling of the damping of the fundamental flexural phonon can be ascribed to the scaling of the fluid dissipation.

Next, we examine the fluid dissipation by looking at the response of the hydrodynamic force of the fluid on the SWCNT resonator.

### Fluid dissipation

From the last section, it can be seen that the fluid dissipation $\left( {Q^{ - 1}} \right)_{{\bf{flu}}}^{th}$ in Eq. (2) depends on the quantities *ω*_1_ and $\tilde c_f\left( {\omega _1} \right)$. With the change in fluid density, the variation of *ω*_1_ is found to be almost negligible compared to $\tilde c_f\left( {\omega _1} \right)$. Thus, the dissipation scaling depends on $\tilde c_f\left( {\omega _1} \right)$. As mentioned before, $\tilde c_f\left( {\omega _1} \right)$ is evaluated from the real Fourier transform of a time-dependent damping kernel *c*_*f*_(*t*). Here, *c*_*f*_(*t*) parameterizes the dissipative part of the hydrodynamic force of the fluid on the SWCNT resonator in response to the fundamental flexural motion (*k* = 1). From the nonequilibrium MD of the ringdown, we can only compute $\tilde c_f(\omega )$ at *ω* = *ω*_1_ but not the time-dependent damping kernel *c*_*f*_(*t*). So, we compute *c*_*f*_(*t*) from equilibrium MD using a formulation based on linear response theory (LRT). In the next subsection, we verify the formulation by carrying out nonequilibrium MD and equilibrium MD simulations on a fluid-coupled resonator system with the resonator atoms frozen, i.e., no phonons (setup I, shown in Fig. 1e). Note that just the fluid is thermally equilibrated in this system. The details on the simulation methodology can be found in the “Materials and methods” section.

#### Resonator with no phonons

LRT^55,57^ states that the response of the thermally equilibrated system to a small external perturbation can be predicted from the thermal fluctuations of the system at equilibrium. In the case of the SWCNT resonator with frozen atoms, flexural motion is set by artificially moving the atoms along the mode shape *ϕ*_1_ with a time dependence as *A*_1_ sin(*ω*_1_*t*). This acts as an external perturbation. During this process, the hydrodynamic force response on the mode, $$\left\langle {P_1} \right\rangle _{neq}$$ can be expressed in terms of equilibrium thermal fluctuations in the hydrodynamic force $$\delta P_1 = P_1 - \left\langle {\delta P_1} \right\rangle _{eq}$$. Here, $$\left\langle . \right\rangle _{neq}$$ and $$\left\langle . \right\rangle _{eq}$$ are nonequilibrium and equilibrium ensemble averages, respectively. Specifically, $$\left\langle {{\mathrm{\Delta }}P_1(t)} \right\rangle _{neq} = {\int}_{ - \infty }^\infty {\kern 1pt} dt\prime \chi _{P_1P_1}\left( {t - t\prime } \right)\lambda \left( {t\prime } \right)$$^57^, where $$\chi _{P_1P_1}(t) =$$$$- \beta \frac{d}{{dt}}\left\langle {\delta P_1(0)\delta P_1(t)} \right\rangle _{eq}$$, *λ*(*t*) = *A*_1_ sin(*ω*_1_*t*), and *β* = (*k*_*B*_*T*)^−1^. This is worked out in details in Supplementary Section 6. We note that $$\left\langle {{\mathrm{\Delta }}P_1(t)} \right\rangle _{neq}$$ can also be parameterized in terms of any *c*_*f*_(*t*) as $$\left\langle {{\mathrm{\Delta }}P_1(t)} \right\rangle _{neq} = {\int}_{ - \infty }^t {\kern 1pt} dt\prime c_f\left( {t - t\prime } \right)\dot \lambda (t\prime )$$. It can then be shown that $$c_f(t) = \beta \left\langle {\delta P_1(0)\delta P_1(t)} \right\rangle _{eq}$$.

**Fig. 5:**
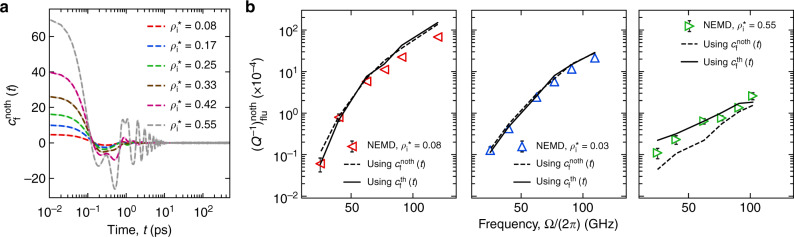
Fluid dissipation using damping kernel. **a**$c_f^{noth}(t)$ versus time at different argon densities for the interior case. **b** A comparison of fluid dissipation $\left( {Q^{ - 1}} \right)_{{\bf{flu}}}^{noth}$ obtained from nonequilibrium molecular dynamics simulation (empty triangles with error bars) with those calculated using the fluid damping kernels, $c_f^{noth}(t)$ (dashed black line) corresponding to the resonator with no-phonon case and $c_f^{th}(t)$ (solid black line) corresponding to the resonator with phonon case. $c_f^{noth}(t)$ and nonequilibrium molecular dynamics simulations in **b** correspond to setup I (shown in Fig. 1e). $c_f^{th}(t)$ computations correspond to setup II (shown in Fig. 1f)

We denote *c*_*f*_(*t*) for the resonator with no phonons as $c_f^{noth}(t)$. Also, we refer *P*_1_(*t*) as $\left( {P_1} \right)_{{\bf{flu}}}^{noth}$. The fluid damping kernel, $c_f^{noth}(t)$ is calculated for setup I (Fig. 1e) at equilibrium for different interior argon densities. The plot is shown in Fig. 5a. The initial value, $c_f^{noth}(0)$ increases monotonically with the increase in density. The dependence of $c_f^{noth}(t)$ on time also changes with density. With the increase in density, $c_f^{noth}(t)$ becomes more oscillatory in time which depicts an elastic behavior. This could result from the solid-like structural reorganization of the argon due to confinement. Similar to Eq. (2), $c_f^{noth}(t)$ is used to calculate the inverse quality factor as a function of frequency, Ω as $\left( {Q^{ - 1}} \right)_{{\bf{flu}}}^{noth} = {\mathrm{\Omega }}(\tilde c)_f^{noth}({\mathrm{\Omega }}){\mathrm{/}}\left( {\xi _1m^0\left( {\omega _1^0} \right)^2} \right)$. Here, $(\tilde c)_f^{noth}({\mathrm{\Omega }})$ is the real Fourier transform of $c_f^{noth}(t)$ at Ω_1_. $\left( {Q^{ - 1}} \right)_{{\bf{flu}}}^{noth}$ is calculated for a range of frequencies at $\rho _i^ \ast$ = 0.08, 0.33, and 0.55. The dissipation at these three densities is also obtained from nonequilibrium MD simulations as outlined in the “Materials and methods” section. A comparison of the dissipation, $\left( {Q^{ - 1}} \right)_{{\bf{flu}}}^{noth}$ obtained from the two different methods is shown in Fig. 5b. A good agreement confirms that the fluid damping kernel, $c_f^{noth}(t)$ formulated using LRT can capture the dissipative response of the fluid.

The simulation setup I with the resonator atoms frozen is used to verify the formulation of the damping kernel. However, the computed fluid damping kernel, $c_f^{noth}(t)$ does not capture the effect of thermal motion or phonons of the resonator at the interface. In the next section, we describe why LRT cannot be used to compute *c*_*f*_(*t*) for the actual system and how another simulation setup, setup II (shown in Fig. 1f) is used to capture those effects in terms of *c*_*f*_(*t*).

#### Resonator with phonons

In the actual case, the SWCNT resonator is vibrating in the fundamental flexural mode (*k* = 1) and all other phonons (*k* = 2, …, 3*N*) are also present in the system due to the thermal motion of the atoms. The thermal motion of the SWCNT atoms can influence the hydrodynamic force response by the fluid, and consequently *c*_*f*_(*t*). However, in this case, we cannot directly apply the LRT and calculate *c*_*f*_(*t*) from equilibrium fluctuations of $\left( {P_1} \right)_{{\bf{flu}}}^{th}\left( t \right)$, where $\left( {P_1} \right)_{{\bf{flu}}}^{th}(t)$ is the modal hydrodynamic force for the resonator with phonons. This is because the external perturbation *λ*(*t*), defined previously, lies in the phase-space of the Hamiltonian of the SWCNT resonator with phonons, at thermal equilibrium^57^. More precisely, *λ*(*t*) coincides with *q*_1_(*t*). Considering this, a workaround would be to suppress the fundamental flexural phonon and allow other phonons in the SWCNT during the equilibrium simulation. This is achieved by attaching a stiff spring (*k*_stiff_ → ∞) at the mid-section along the length of the SWCNT (setup II, shown in Fig. 1f). The stiff spring keeps the midsection at rest and thus suppresses the fundamental flexural modes (*k* = 1, 2). In this scenario, LRT can be used to calculate *c*_*f*_(*t*) for the SWCNT resonator, corresponding to a perturbation along *ϕ*_1_. Although the aforementioned fix affects the nature of few other phonons, the calculated *c*_*f*_(*t*) can effectively capture the influence of the thermal motion of the SWCNT atoms at the fluid–structure interface. For the resonator with phonon case, we denote *c*_*f*_(*t*) as $c_f^{th}(t)$.

**Fig. 6:**
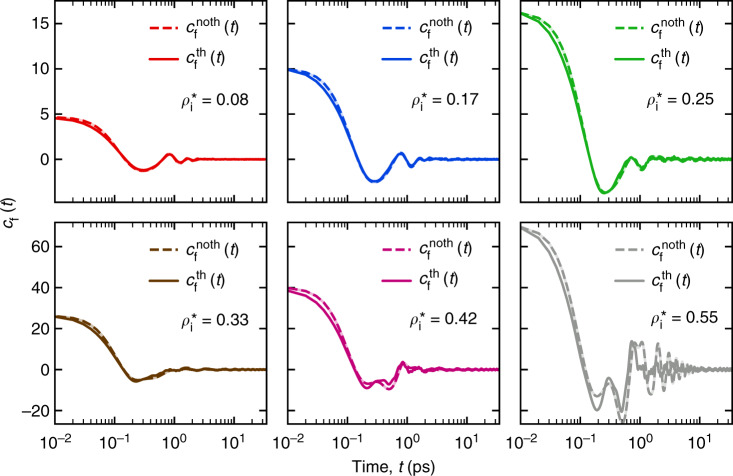
**A comparison between*c***_***f***_**(*t*) computed for a SWCNT resonator with phonons (solid line) and with no phonons (dashed line) for different interior argon densities**

Figure 6 shows the comparison of *c*_*f*_(*t*) between the resonator with phonon case and the resonator with no-phonon case at different interior argon densities. The initial values, *c*_*f*_(0) of both the cases are approximately equal. In terms of the time dependence of *c*_*f*_(*t*), the two cases differ increasingly as we go towards higher densities. Especially for the highest density ($\rho _i^ \ast = 0.55$), the resonator with no-phonon case displays more oscillations in the kernel at longer times whereas the resonator with phonon case does not. If the oscillations in the no-phonon case resulted from solid-like structural organizations due to nanoconfinement, then the thermal motion or phonons tend to break those organizations, and consequently suppress the solid-like response behavior. Ultimately, we are interested, if the absence or presence of phonons in the resonator reflects in the fluid dissipation. To verify this, we can compute the fluid dissipation for the resonator with no-phonon cases using the damping kernel corresponding to the phonon cases, i.e., $c_f^{th}(t)$. The plots are shown in Fig. 5b. It can be seen that except for the highest density case $\left( {\rho _i^ \ast = 0.55} \right)$, the fluid dissipation estimated using $c_f^{noth}(t)$ (dashed lines) and $c_f^{th}(t)$ (solid lines) are almost overlapping. The only difference in the fluid dissipation is for the highest density case which can be ascribed to the difference in the time dependence of *c*_*f*_(*t*) at longer times (Fig. 6, last panel). This suggests that, for low to moderately high densities $\left( {\rho _i^ \ast \lesssim 0.4} \right)$, the fluid dissipation, which actually results from the oscillatory fluid flow due to the resonant motion of the structure is not affected by its interfacial atomic thermal motion or phonons. Specifically, (*Q*^−1^)_**sys**_ at lower densities can be approximated as $\left( {Q^{ - 1}} \right)_{{\bf{sys}}} = \left( {Q^{ - 1}} \right)_{{\bf{int}}}^v + \left( {Q^{ - 1}} \right)_{{\bf{flu}}}^{noth}$. Next, we use $c_f^{th}$ to examine different cases of confined fluid damping under the fundamental flexural motion of the resonator.

#### Scaling of confined fluid damping

**Fig. 7:**
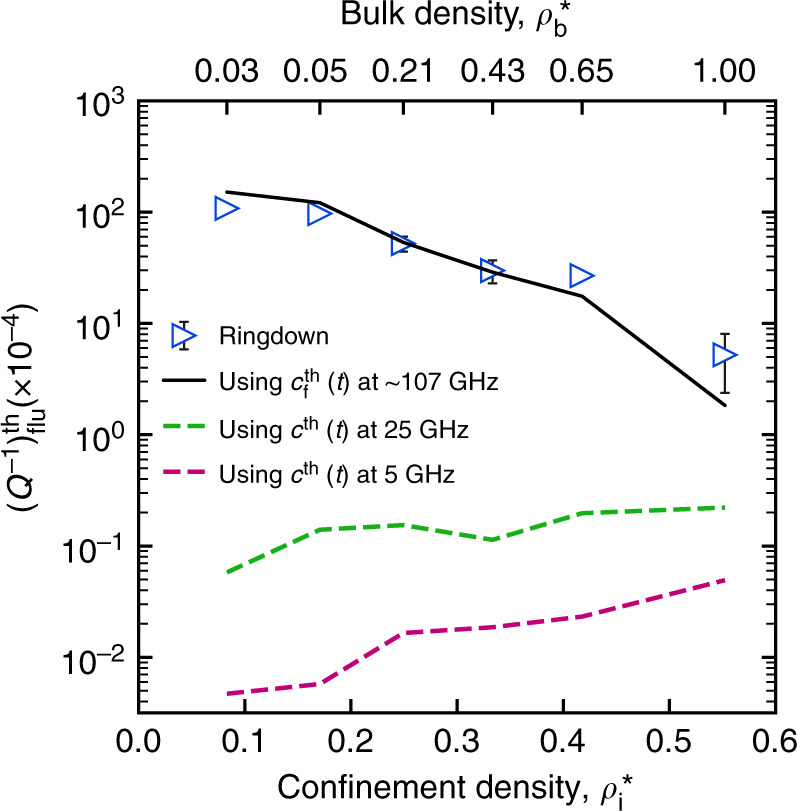
Density scaling of fluid dissipation using damping kernel. A comparison of fluid dissipation obtained from ringdown simulation (empty triangles with error bars) with that computed using $c_f^{th}(t)$ (black solid line) at the resonant frequencies ω_1_. Using $c_f^{th}(t)$, the fluid dissipation scaling is also computed at two other frequencies: 25 GHz (green dashed line) and 5 GHz (magenta dashed line). All the cases correspond to the SWCNT with interior argon. For argon, *ρ* = ρ^*^ × 1687.5 kg/m^3^

Having computed $c_f^{th}(t)$ at different densities of interior argon for the actual system, we can estimate $\left( {Q^{ - 1}} \right)_{{\bf{flu}}}^{th}$ at any frequency Ω similar to the no-phonon case. First, we estimate $\left( {Q^{ - 1}} \right)_{{\bf{flu}}}^{th}$ at the resonant frequency *ω*_1_ of the fundamental flexural mode at all interior argon densities. The comparison is shown in Fig. 7. A good agreement clearly reveals that the inverse scaling of the fluid damping is directly related to the fluid response behavior under confinement. This fluid response under confinement is captured by $c_f^{th}(t)$. The time dependence of $c_f^{th}(t)$ signals a frequency-dependent scaling of the confined fluid dissipation. We examine this by estimating $\left( {Q^{ - 1}} \right)_{{\bf{flu}}}^{th}$ at two additional frequencies, 25 and 5 GHz which are lower than the resonant frequency *ω*_1_ of the actual system. The frequencies may correspond to SWCNT resonator of much larger length (>20 nm) with the same radius. The scaling is shown in Fig. 7. It can be seen that the scaling behavior changes at different frequencies. At the intermediate frequency, i.e., 25 GHz, the dissipation scaling is nonmonotonic and at the lower frequency, i.e., 5 GHz, the fluid dissipation scales directly with the increase in density. The frequency-dependent scaling behavior can be attributed to a coupled effect of the viscoelasticity and nanoscale confinement of the fluid. It should also be noted that a ~5-fold reduction in frequency leads to an ~100-fold reduction in fluid dissipation. Thus, a high-quality factor can be achieved.

## Discussion

In this section, we discuss our findings in the light of previous studies on fluid damping in fluid channel-based resonators and intrinsic damping of flexural phonons in 1D and 2D resonators. The nonmonotonicity in fluid dissipation with density or viscosity of the fluid was first observed in microchannel resonators^44^ with confinement length (*l*_*c*_) in ~1–10 μm and resonant frequency (*f*_1_) in ~100–500 of KHz. A formulation using classical NS equation with no-slip boundary condition by Sader et al.^58^ could quantify the fluid dissipation and nonmonotonic scaling. The nonmonotonicity was also observed in the case of nanochannel resonators with *l*_*c*_ ~ 100–700 nm and *f*_1_ ~ 0.5–25 MHz^42,43^. However, in these nanochannel resonators the fluid dissipation could not be quantitatively explained using the formulation by Sader et al. This could be due to slip-effects at the fluid–structure interface and frequency dependence of slip length and viscosity^46^ which can be expected in our argon filled SWCNT system with *l*_*c*_ ~ 1.3 nm and *f*_1_ ~ 107 GHz. Though Sader et al. model can be corrected by incorporating these effects under a multiscale approach^47^, it is not pursued in this study. Instead, a more direct method is employed where the dissipative behavior of the fluid is parameterized in terms of a damping kernel. Indeed, the fluid dissipation estimated using the damping kernel reveals a frequency dependent scaling behavior in the case of SWCNT with confined fluid.

**Fig. 8:**
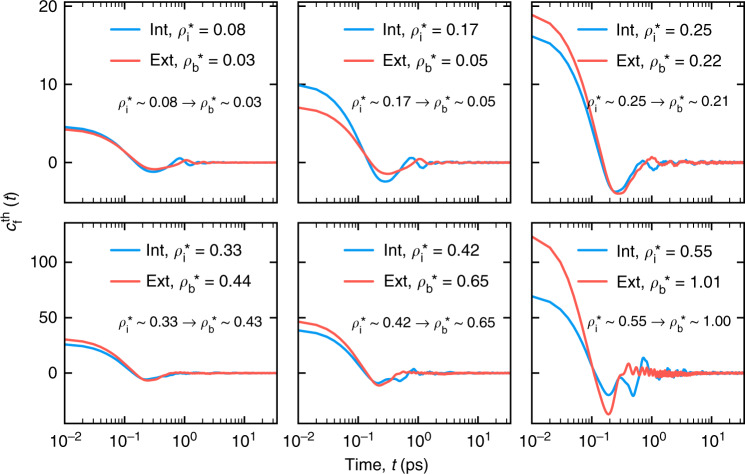
A comparison of $c_f^{th}(t)$ computed for the SWCNT with interior argon (blue line) with that of exterior argon (red line) for different fluid densities. $\rho _b^ \ast$ is the reservoir density required to fill the SWCNT with a confined density $\rho _i^ \ast$ (refer to Figure 1 in Supplementary Section 1)

The scaling for the confined fluid case is considerably different from the fluid exterior case which displays a conventional increase in fluid dissipation with density^36,37^. This prompted in comparing the fluid damping kernel $c_f^{th}(t)$ in the fluid interior and exterior case. Figure 8 shows the comparison. Each panel in Fig. 8 displays $c_f^{th}(t)$ for SWCNT coupled with argon at some exterior density $\rho _b^ \ast$ and interior density $\rho _i^ \ast$, where $\rho _b^ \ast$ is the reservoir density required to fill the SWCNT with a confined density $\rho _i^ \ast$ (refer to Figure 1b in Supplementary Section 1). The plots reveal that the time dependence of $c_f^{th}(t)$ in the interior case is significantly different from the exterior case. For example, the $c_f^{th}(t)$ in the highest density case (last panel), exhibits a highly dissipative behavior (indicated by the decay in the response) in the exterior case, whereas in the interior case, the dissipative nature of the response is negligible.

The fact that $\left( {Q^{ - 1}} \right)_{{\bf{int}}}^f$ is approximately equal to $\left( {Q^{ - 1}} \right)_{{\bf{int}}}^v$ during ringdown of the fundamental flexural phonon provides some insights on the intrinsic dissipation mechanism. The known intrinsic mechanisms by which the flexural phonon can dissipate energy are nonlinear mode coupling^27,28^, mode hybridization^29^, and Akhiezer mechanism^59^. The nonlinear mode coupling is the coupling of the fundamental mode (*k* = 1) with the rest of the phonon ensemble due to the anharmonic terms in the resonator potential. Under this mode coupling, one way the fundamental mode can dissipate is by directly thermalizing with the rest of the phonons at some decay rate. In another way, the fundamental mode first transfers energy to a group of modes it is nonlinearly coupled with, and the mode group then thermalizes with their respective baths at particular rates. Here, a mode’s bath comprises the rest of the phonon modes and the fluid if fluid is present. Thus, the energy of the fundamental mode decays at a rate which is an average over the direct and the indirect thermalization rates and this phenomenon is called mode hybridization^29^. If the flexural motion associates a strain field in the structure, dissipation due to Akhiezer mechanism can play a role. Under Akhiezer mechanism, the strain coupling due to finite Grüneisen constants perturb the energy of all the phonons and they relax at some microscopic timescales by exchanging energy with their respective baths. Both the decay channels due to mode hybridization and Akhiezer mechanism would be affected by the presence of fluid^60^. Since, we found that $\left( {Q^{ - 1}} \right)_{{\bf{int}}}^f \approx \left( {Q^{ - 1}} \right)_{{\bf{int}}}^v$, we infer that dissipation due to mode hybridization and Akhiezer mechanism is not significant. This could be true as the amplitude considered during the ringdown process is reasonably small^29,59^.

## Conclusions

In conclusion, we report the mechanical energy dissipation during the fundamental flexural resonance of an SWCNT resonator coupled with interior and exterior argon. Our dissipation calculations involve ringdown simulations using molecular dynamics (MD). In the fluid-coupled resonator, we identify the two sources of dissipation as phonon-mediated intrinsic dissipation and extrinsic dissipation due to the fluid. The intrinsic dissipation results from the anharmonic coupling and direct thermalization of the fundamental flexural phonon with the rest of the phonon ensemble. The intrinsic dissipation under this mechanism remains unaffected by fluid interactions. The fluid dissipation component contributes to the unexpected scaling of the net dissipation with density in the fluid interior case. To understand the mechanism underlying the unexpected behavior, a fluid damping kernel is formulated using the LRT. The fluid damping kernel captures the dissipative response of the fluid to the flexural motion. The magnitude of fluid dissipation is shown to be accurately quantifiable using the fluid damping kernel. The kernel is used to examine the effect of thermal motion of the resonator atoms (or phonons) on the fluid dissipation. It is found that the thermal motion does not affect till moderately high fluid densities $\left( {\rho _i^ \ast \lesssim 0.4} \right)$. The fluid dissipation computed using the kernel can reproduce the unexpected scaling as obtained from ringdown simulation for interior argon cases. Comparing the fluid damping kernel for the exterior and interior case, it becomes apparent that the hydrodynamic response of the fluid under a nanoconfinement is indeed different from a bulk fluid. Our finding of extremely low dissipation in nanoconfined fluids can eventually solve the long-standing challenge of high-precision sensing of analytes from dense fluids.

## Materials and methods

A (10, 10) SWCNT of length (*l*_*z*_) 20 nm is constructed which forms the resonating structure. The structure is made of 3280 carbon atoms and resembles a tube (as shown in Fig. 1a) with an approximate radius (*r*) of 0.68 nm. Argon is considered as the fluid medium. The interaction between the resonating structure and the fluid is studied in two different cases: (i) SWCNT with interior fluid and (ii) SWCNT with exterior fluid. In the case of SWCNT with interior fluid (shown in Fig. 1b), all the fluid atoms (or molecules) were placed inside the tube. A periodic boundary condition is applied in the axial direction of the tube to keep the fluid confined during the simulation. In the case of SWCNT with exterior fluid (shown in Fig. 1c), the fluid is placed outside the tube. A large enough simulation box (with dimensions *l*_*x*_ × *l*_*y*_ × *l*_*z*_) is defined to surround the fluid outside SWCNT in the radial direction and a periodic boundary condition is applied in all the three directions. The bonded interaction between the carbon atoms in the SWCNT is described by the modified Tersoff potential^61^. Two-body Lennard–Jones (LJ) potential^62^ is used to model the non-bonded interaction between the argon atoms of the fluid and between the argon atoms of the fluid and the carbon atoms of the SWCNT.

We use the LAMMPS package^63^ for the MD simulations. The first stage of simulations involves preparing the equilibrated samples of the fluid–resonator system for a range of fluid densities. The SWCNT is pre-stretched by 5% to avoid any buckling. Two rings of atoms, which correspond to 40 carbon atoms at each end of the SWCNT, are kept frozen to impose a clamped boundary condition. The thermal motion is allowed in rest of the (*N*) SWCNT atoms. Then, the argon atoms are placed at a pre-defined spacing in the cylindrical region inside the SWCNT in case of interior fluid and in the region between the cylindrical SWCNT and the cuboidal simulation box in case of exterior SWCNT. A different number of argon atoms are fitted inside those regions by altering the spacing, which results in different densities. The case with no argon atoms corresponds to an SWCNT resonator in the vacuum. A non-dimensional density can be calculated in each case as $\rho _\lambda ^ \ast = M\sigma ^3{\mathrm{/}}V_\lambda$ where *λ* is *i* or *b*, corresponding to the interior and exterior fluid case respectively, *M* is the number of fluid atoms in the system, *σ* is its LJ diameter, and *V*_*λ*_ is the volume of space available for the fluid, which is *V*_*i*_ = *πr*^2^*l*_*z*_ in case of interior fluid and *V*_*b*_ = (*l*_*x*_*l*_*y*_*l*_*z*_ − *πr*^2^*l*_*z*_) in case of exterior fluid, respectively. The fluid and the SWCNT atoms are, then, assigned some initial velocity drawn from a Gaussian distribution corresponding to a temperature, *T* (=300 K) and integrated under NVT^64^ ensemble for 5 ns to reach thermal equilibrium. The final structure of SWCNT with interior and exterior fluid is shown in Fig. 1b, c, respectively.

To compare the interior and the exterior fluid cases, we reference the density of the interior fluid in terms of density and pressure of the bulk reservoir corresponding to the exterior fluid case. For this, we set up separate simulations of SWCNT inside a bulk argon reservoir (shown in Fig. 1d) and carry out SWCNT filling and equilibration. A considerably smaller SWCNT is considered in these simulations to reduce the computational cost. Different densities are achieved by controlling the pressure of the fluid in the reservoir under NPT^65^ ensemble, with the temperature set to 300 K. The simulation box of the reservoir adjusts its dimension to equilibrate at a particular fluid density for a given pressure. A relation of the bulk pressure with the bulk density $\left( {\rho _b^ \ast } \right)$ and the interior density $\left( {\rho _i^ \ast } \right)$ is obtained from these simulations.

The dissipation study involves ringdown measurements^28,66^ using non-equilibrium MD simulations. The equilibrated samples form the starting configuration for the non-equilibrium MD simulations. In the equilibrated samples, all the phonon modes of the SWCNT resonator share the same thermal energy equal to *k*_*B*_*T* where *k*_*B*_ is the Boltzmann constant and *T* is the temperature. Ringdown is carried out by imparting some extra energy to the fundamental flexural phonon (*ϕ*_1_) of the SWCNT. The mode shape, *ϕ*_1_ corresponds to one of the two degenerate fundamental flexural phonons of the doubly-clamped SWCNT resonator and is obtained using real space quasiharmonic (QHMR) method (see Supplementary Section 2). In MD, the extra energy is imparted by rescaling the velocities of the SWCNT atoms from the starting configuration. If the velocities of the SWCNT atoms are denoted by *v*_*i*,*α*_ in the equilibrated starting configuration, then the velocity perturbation along *ϕ*_1_ leads to $v_{i,\alpha } \leftarrow v_{i,\alpha } + \epsilon _v\phi _1^{i,\alpha }$. Here, *ϕ*_1_ (shown in Fig. 2b) represents the shape of the phonon mode which is a vector of length 3*N* where *N* is the number of SWCNT atoms in thermal motion and 3 corresponds to their degrees of freedom. For any atom indexed as *i* and *α* = 1, 2, and 3, corresponding to the *x*, *y*, and *z* directions, respectively, $\phi _1^{i,\alpha }$ is the contribution of *i*th atom to the mode shape along *α*th direction. The scaling parameter $\epsilon _v$ is kept small such that the change in thermal energy of the sample due to the perturbation is within 0.7% of the initial thermal energy of the system, i.e., 3*Nk*_*B*_*T*. Following the velocity rescaling, both the SWCNT and the fluid are evolved under constant energy (NVE) ensemble as an isolated system. During this ringdown process, the extra energy of the fundamental flexural phonon is redistributed among all other phonon modes and fluid atoms. Consequently, the displacement, *q*_1_(*t*) and the velocity, $\dot q_1\left( t \right)$ of the fundamental flexural phonon exhibit a decaying oscillatory nature. The decay in the displacement amplitude of *q*_1_(*t*) (shown in Fig. 2d) can be associated with a time scale $\tau _1^d$, called the damping time. From $\tau _1^d$, the inverse quality factor, which is a non-dimensional measure of dissipation, can be calculated as $Q^{ - 1} = 2{\mathrm{/}}\left( {\omega _1\tau _1^d} \right)$ (see Supplementary Section 4e), where *ω*_1_ is the angular frequency of the fundamental flexural phonon obtained by taking fast Fourier transform of the velocity history, $\dot q_1(t)$.

For the purpose of dissipation analysis, we consider two more simulation setups of the fluid-coupled resonator system; setup I and setup II. In case of setup I (shown in Fig. 1e), the thermal motion of all the resonator atoms is kept frozen throughout the simulations. Under this condition, the equilibrium simulations entail updating the positions and velocities of just the fluid atoms during NVT or NVE integration. The dissipation study under nonequilibrium simulations involves deforming the resonator atoms along *ϕ*_1_ by artificially setting their position at each step and evolving the fluid atoms by NVE integration. If the positions of the frozen SWCNT atoms are denoted by *x*_*i*,*α*_ before the start of the nonequilibrium MD simulation, then their position, *x*_*i*,*α*_ at any instant during the dissipation study is set as $x_{i,\alpha }(t) = x_{i,\alpha }^0 + A{\kern 1pt} {\mathrm{sin}}({\mathrm{\Omega }}t)\phi _1^{i,\alpha }$. Here, *A* corresponds to the values obtained from the ringdown simulation of the actual system. In the actual system, the fundamental flexural resonance occurs at a fixed frequency *ω*_1_. In setup I with artificially imposed deformation, we have control over the deformation frequency, Ω. Ω is varied over a range of frequencies, including *ω*_1_ corresponding to the actual system. Due to fluid dissipation, the energy of the system increases with the periods of deformation. The amount of dissipation per period, *D* is calculated from the slope of the average energy of the system with the number of periods (shown in Fig. 2f). Using *D*, the inverse quality factor is calculated as *Q*^−1^ = *D*/(2*πE*_sto_). Here, *E*_sto_ is the maximum energy stored during the prescribed deformation. The simulation setup corresponding to setup II (shown in Fig. 1f) involves attaching stiff springs (*k*_stiff_ → ∞) to 160 carbon atoms at the mid-section of the resonator. The purpose is to restrict these atoms from moving in any of the three directions. Setup II is used to carry out equilibrium simulations only.

## Supplementary information

Supplementary Information
