# Supplementary material for: Anomalous scaling of flexural phonon damping in nanoresonators with confined fluid
Source: Microsyst Nanoeng. 2019 Jan 14;5:2. doi: 10.1038/s41378-018-0041-2 (PMC6330506; doi:10.1038/s41378-018-0041-2)
Supplement: Supplementary file 1 — Supplementary Information [file 41378_2018_41_MOESM1_ESM.pdf]

Supplementary Information: Anomalous scaling of flexural phonon damping in nanoresonators with confined fluid

Subhadeep De[1,a)] and Narayana R. Aluru[1,2]

[1)] *Department of Mechanical Science and Engineering*

[2)] *Beckman Institute for Advanced Science and Technology, University of Illinois at Urbana-Champaign, Urbana, Illinois 61801, United States.*

[a)] Electronic mail: sde4@illinois.edu

S.1. RESERVOIR DENSITY (ρ_b^*) AND CONFINEMENT DENSITY (ρ_i^*)

Figure 1a shows the change in bulk density, ρ_b^* of argon as the pressure in the reservoir is increased between $\sim 10 - 10^4$ bars. For the pressure range considered in this study, argon exists either in the vapor or the supercritical state[1]. For any higher pressure, argon turns solid and will prevent the filling of the SWCNT. A cubic spline fit is performed to the data points obtained from equilibrium MD simulations to relate the pressure and the density, ρ_b^* at any intermediate point in the range. Corresponding to the reservoir pressure (or density) range, the confinement density, ρ_i^* of argon in the filled SWCNT varies between $\sim 0.01 - 0.57$ as shown in Figure 1b. It can be seen that ρ_i^* increases with ρ_b^* (or pressure) linearly with the linear slope changing at $\rho_i^* \sim 0.20$. A bilinear fit is used to capture the relation. The fit can be used to obtain the bulk density corresponding to any confinement density or vice versa. This is later used to plot and compare the interior and exterior cases of dissipation in terms of the density.

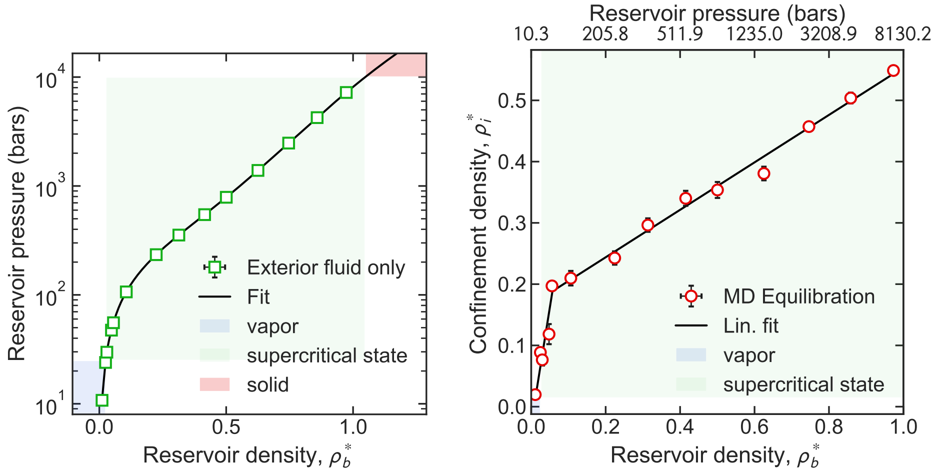

(a) (b)

FIG. 1. (a) Bulk pressure versus bulk density, ρ_b^* of argon in the reservoir. (b) Confinement density, ρ_i^* of argon in the SWCNT versus bulk density, ρ_b^* (lower X-axis) and bulk pressure (upper X-axis) of argon in the reservoir. For argon, $\rho = \rho^* \times 1687.5$ kg/m^3. The green squares with error bars (extremely small and not visible) in (a) and the red circles with error bars in (b) are the data points from equilibrium molecular dynamics (MD) simulations. In (a) and (b), the black line is a cubic spline fit and bilinear fit, respectively, to the equilibrium MD data. The light shades of blue, green and red in the background indicate the states of argon, which are vapor, supercritical, and solid, respectively.

S.2. COMPUTATION OF PHONON MODE SHAPES

The mode shapes of the doubly-clamped SWCNT resonator can be obtained by carrying out real space quasiharmonic (QHMR) analysis[2,3] of the equilibrated SWCNT structure. Using QHMR analysis, for a structure with N atoms, a mass-weighted $3N \times 3N$ force constant matrix, $\mathbf{\Phi}$ can be defined with its elements given by $\mathbf{\Phi}_{3j-3+\alpha,3k-3+\beta} = \frac{1}{\sqrt{M_j M_k}} \frac{\partial^2 V(\mathbf{x})}{\partial x_{j,\alpha} \partial x_{k,\beta}}\Big|_{\mathbf{x}=\mathbf{x}^0}$, where V is the potential energy, j and k are the atom indices, α and β are the directions, M_j and M_k are the atomic masses, $\mathbf{x} = (\vec{x}_1, \ldots, \vec{x}_N)$ denotes the instantaneous positions of all the atoms in the system, $x_{j,\alpha}$ and $x_{k,\beta}$ are the components of the instantaneous position vectors $\vec{x}_j$ and $\vec{x}_k$, respectively, along the αth and β^{th} directions and the superscript '0' is used to denote equilibrium configuration. The eigenvectors of the matrix, $\mathbf{\Phi}$ correspond to the phonon mode shapes, ϕ_k for $k = 1$ to $3N$, k being the mode index. From the eigenvalues, λ_k of $\mathbf{\Phi}$, the phonon frequencies can be calculated as $\omega_k = \sqrt{\lambda_k}$. If the phonon frequencies are sorted in an increasing order, it can be found that the two lowest frequencies (ω_1 and ω_2) are approximately equal, i.e., $\omega_1 \sim \omega_2$ such that $k = 1$ and $k = 2$ are the two degenerate fundamental flexural phonons with mode shapes ϕ_1 and ϕ_2. Due to clamping of the resonator at both ends, the Born-von Karman boundary condition[4] cannot be applied and the $3N \times 3N$ force constant matrix, $\mathbf{\Phi}$, in the real space cannot be reduced to a more conventional $3B \times 3B$ dynamical matrix in the reciprocal space using Bloch's theorem[5], where B is the number of basis atoms in the lattice structure of SWCNT. The large size of the force constant matrix makes the QHMR analysis computationally expensive.

S.3. REAL SPACE TO PHONON SPACE

The resonant motion of any phonon mode, k, of the structure involves the collective movement of all of its atoms. To understand the motion, a coordinate transformation using the phonon mode shapes, ϕ_k is required, from the real space to the phonon space, which can be written as

$$S_k = \sum_{\alpha=1}^{3} \sum_{i=1}^{N} s_{i,\alpha} \phi_k^{i,\alpha}.$$

Here, $s_{i,\alpha}$ can be displacement, velocity or force corresponding to the ith resonator atom along the αth direction in the real space coordinate system, where α takes values 1, 2, or 3 corresponding to the x, y, or z directions, and S_k corresponds to the same quantity (displacement, velocity or force) for the kth mode in the modal space coordinate. Following this transformation, at any instant for some mode k, the displacement of the phonon mode (q_k), the velocity of the phonon mode ($\dot{q}_k$) and the hydrodynamic force by the fluid on the phonon mode ($(P_k)_{\mathbf{flu}}$) can be expressed as

$$
\begin{aligned}
q_k &= \sum_{\alpha=1}^{3}\sum_{i=1}^{N} u_{i,\alpha}\phi_k^{i,\alpha}, \\
\dot{q}_k &= \sum_{\alpha=1}^{3}\sum_{i=1}^{N} v_{i,\alpha}\phi_k^{i,\alpha}, \\
(P_k)_{\mathbf{flu}} &= \sum_{\alpha=1}^{3}\sum_{i=1}^{N} (F_{i,\alpha})_{\mathbf{flu}}\phi_k^{i,\alpha}.
\end{aligned}
$$

Here, u and v and $(F)_{\mathbf{flu}}$ is displacement, velocity, and hydrodynamic force, respectively, on the resonator atom i along the αth direction. $k = 1, \ldots, 3N$ corresponding to the $3N$ phonon modes and $k = 1$ is the fundamental flexural mode.

S.4. DAMPED HARMONIC OSCILLATOR MODEL

A. Amplitude

The phonon modes obtained from the real space quasiharmonic (QHMR) analysis[2,3] are usually orthonormalized, i.e., the inner product, $\bar{\phi}_k\bar{\phi}_l = \delta_{kl}$, where $\bar{\phi}_k$ and $\bar{\phi}_l$ are the kth and l^{th} modal vectors (or modeshapes) and δ_{kl} is the Kronecker delta function. The amplitude of any phonon mode obtained using the orthonormalized modal vector may not be its true measure of amplitude. Conventionally, the amplitude of oscillation of a resonator is measured at the point of maximum displacement in its volume. The oscillation amplitude of that point is regarded as the amplitude of the resonator. If the modeshape ϕ_k is orthonormalized and its magnitude is maximum at the i_m^{th} atom along the α_m^{th} direction, then corresponding to any amplitude A_k^o of the displacement $q_k(t)$ of the orthonormalized mode ϕ_k, its true measure

of amplitude, A_k is given by $A_k = A_k^o|\phi_k^{i_m,\alpha_m}|$. Here, $\phi_k^{i_m,\alpha_m}$ is the component of the modal vector, ϕ_k evaluated at the i_m^{th} atom along the α_m^{th} direction.

B. Effective mass

A resonator being an extended object, one can choose to record the displacement at any point along its volume, and consequently, the amplitude of resonance is not defined uniquely. However, the system's potential energy is a unique quantity and should be independent of the measurement criteria. Thus, the mass parameter in the damped harmonic oscillator model is defined to be the effective mass of the resonator where the effective mass is an adjustment over the true mass of the resonator to ensure that the potential energy of the motion is uniquely defined for the system[6].

Here, a derivation of the effective mass of a resonator is shown corresponding to the assumption that the amplitude of oscillation of the resonator is measured at the point of maximum displacement in its volume. The effective mass μ_k corresponding to any phonon mode k can be used to calculate the maximum potential energy stored by the mode as $(U^p)_{max} = \mu_k \omega_k^2 A_k^2/2$. μ_k is a fraction (ξ_k) of total mass m of the resonator with N atoms as $\mu_k = \xi_k m$. The maximum potential energy can also be calculated as sum of the maximum potential energies $(U_i^p)_{max}$ stored by each atom (with mass m/N) during vibration under the mode shape ϕ_k. $(U_i^p)_{max}$ can be expressed as $(U_i^p)_{max} = \sum_{\alpha=1}^3 (m/N)\omega_k^2 |\phi_k^{i,\alpha} A_k^o|^2/2 = m\omega_k^2 A_k^2/(2N|\phi_k^{i_m,\alpha_m}|^2)\sum_{\alpha=1}^3 |\phi_k^{i,\alpha}|^2$, where $\alpha = 1, 2$ and 3 corresponds to the x, y and z directions, and A_k^o is substituted as $A_k^o = A_k/|\phi_k^{i_m,\alpha_m}|$ in the last step. Summing $(U_i^p)_{max}$ over all the N atoms, we get

$$\begin{aligned}
\sum_i^N (U_i^p)_{max} &= m\omega_k^2 A_k^2/(2N|\phi_k^{i_m,\alpha_m}|^2)\sum_i^N \sum_{\alpha=1}^3 |\phi_k^{i,\alpha}|^2 \\
&= m\omega_k^2 A_k^2/(2N|\phi_k^{i_m,\alpha_m}|^2)\bar{\phi}_k \bar{\phi}_k \\
&= m\omega_k^2 A_k^2/(2N|\phi_k^{i_m,\alpha_m}|^2)\delta_{kk} \\
&= m\omega_k^2 A_k^2/(2N|\phi_k^{i_m,\alpha_m}|^2).
\end{aligned}$$

Equating $(U^p)_{max}$ with $\sum_i^N (U_i^p)_{max}$, we get, $\mu_k = m/(N|\phi_k^{i_m,\alpha_m}|^2)$ or $\xi_k = 1/(N|\phi_k^{i_m,\alpha_m}|^2)$.

C. Equation of motion

During flexural vibration of long slender structures like a carbon nanotube, nonlinearity is prevalent. The nonlinear mode coupling effect on the fundamental flexural mode of an isolated SWCNT can be effectively represented by introducing a damping term and an amplitude dependent frequency term in the harmonic oscillator model of the flexural motion. The presence of fluid additionally contributes to the damping. In this context, since we have restricted our study to smaller amplitudes, the damping and the frequency parameter can be treated as average quantities constant over the motion. Thus, a damped harmonic oscillator model is sufficient to describe the ringdown of the flexural mode as

$$\mu_k \ddot{q}_k + \mu_k \nu_k \dot{q}_k + k_k q_k = 0, \tag{1}$$

where q_k represents the displacement of the flexural phonon. μ_k, k_k, and ν_k are the effective mass, effective stiffness, and damping coefficient, respectively of the phonon mode. For an empty SWCNT in vacuum, $\mu_k = \xi_k m$ (refer Sec. S.4 B), where m is the mass of the resonator and ξ_k is a fraction (< 1) which depends on the mode shape. Also, $k_k = \mu_k(\omega_k^*)^2$ where ω_k^* is the resonant frequency of the phonon mode in a case where there is no damping, and is related to its resonant frequency ω_k in the present case with damping as $\omega_k = \sqrt{(\omega_k^*)^2 - \frac{\nu_k^2}{4}}$. The damping coefficient ν_k and the inverse quality factor Q_k^{-1} are related as $\nu_k = \omega_k Q_k^{-1}$ (refer Sec. S.4 E). The fundamental flexural phonon is represented by $k = 1$. In the case of empty SWCNT in vacuum, it is found that $Q^{-1} \ll 1$, which implies $\omega_1 \gg \nu_1/2$. Consequently, $\omega_1 \sim \omega_1^*$ and k_1 can be written as $k_1 = \xi_1 m(\omega_1)^2$. We represent m and ω_1 in the case of empty SWCNT in vacuum as m^0 and ω_1^0, respectively. When the SWCNT is coupled with the interior or exterior argon, the effective mass of the flexural phonon can change due to the added mass by the fluid. However, fluid coupling doesn't alter the effective stiffness of the phonon mode. Considering this and accounting for the damping, the resonant frequency of the flexural phonon can be expressed as[7]

$$\omega_1 = \sqrt{\frac{m^0}{m^0 + \Delta m}(\omega_1^0)^2 - \frac{\nu_1^2}{4}}, \tag{2}$$

where Δm is the added mass due to the fluid during the fundamental flexural motion.

D. Damping time

The solution to the equation of motion (Eq. 1) is $q_k(t) = A_k e^{-\nu_k t/2}\cos(\omega_k t - \theta)$. Here, A_k is the initial amplitude, and $A_k e^{-\nu_k t/2}$ denotes the time decay of the initial amplitude. The exponential decay can be characterized by a damping time τ_k^d as $\tau_k^d = 2/\nu_k$.

E. Quality factor

The total energy of the oscillator at any instant is $E_k = \mu_k \dot{q}_k^2/2 + \mu_k(\omega_k^*)^2 q_k^2/2$, where μ_k is the mass of the oscillator. Using the equation of motion, the rate of change of energy can be expressed as $dE_k/dt = -\mu_k\nu_k\dot{q}_k^2$. The energy lost during a period of oscillation will be

$$\begin{aligned}\Delta E_k &= -\int_{\theta/\omega_k}^{(2\pi+\theta)/\omega_k} dt\,(dE/dt)\\ &= \mu_k\nu_k A_k^2 \int_{\theta/\omega_k}^{(2\pi+\theta)/\omega_k} dt\, e^{-\nu_k t}\left[\nu_k/2\cos(\omega_k t-\theta)+\omega_k\sin(\omega_k t-\theta)\right]^2 .\end{aligned}$$

Under weak damping ($\nu_k \ll 2\omega_k^*$), the exponential factor in the integral can be approximated as unity ($e^{-\nu_k t/2} \sim 1$) and consequently carrying out the integration would lead to $\Delta E_k = \pi\mu_k\nu_k A_k^2(\omega_k^2+\nu_k^2/4)/\omega_k = \pi\mu_k(\omega_k^*)^2 A_k^2(\nu_k/\omega_k)$. The maximum energy stored by the oscillator is at the initial instant as $E = \mu_k(\omega_k^*)^2 A_k^2/2$. The inverse quality factor, defined as the ratio of energy dissipated over an oscillation period to the maximum energy stored, can thus be calculated as $Q_k^{-1} = \Delta E_k/(2\pi E_k) = \nu_k\omega_k = 2/(\tau_k^d\omega_k)$.

F. Relaxation time

The phonon relaxation time, $(\tau_k)_{ph}$ denotes the decay time of the energy of the phonon modes. From the expression of rate of change of energy, $dE_k/dt = -\mu_k\nu_k\dot{q}_k^2 = e^{-\nu_k t}(\ldots)$, $(\tau_k)_{ph}$ can be approximated as $(\tau_k)_{ph} = 1/\nu_k = \tau_k^d/2$.

S.5. FLUID DAMPING CONSTANT FROM HYDRODYNAMIC FORCE

The hydrodynamic force, $(P_1)^{th}_{\mathbf{flu}}$ on the fundamental flexural mode ($k = 1$) can be calculated by projecting the hydrodynamic force, $(F_{i,\alpha})_{\mathbf{flu}}$ on each resonator atom along the mode shape ϕ_1 (refer Sec. S.3). $(P_1)^{th}_{\mathbf{flu}}$ can be expressed, in the most general way, as the sum of a conservative force proportional to the displacement $q_1(t)$ of the flexural phonon and a damping force with memory, proportional to the velocity $\dot{q}_1(t)$, such that[8]

$$\langle (P_1)^{th}_{\mathbf{flu}}(t)\rangle_{neq} = -k_f \langle q_1(t)\rangle_{neq} - \int_{-\infty}^{t} c_f(t-t')\langle \dot{q}_1(t')\rangle_{neq}\, dt'. \tag{3}$$

Here, $\langle . \rangle_{neq}$ denotes average over different time instants during the nonequilibrium ringdown simulation. We note that $q_1(t)$ at any instant can be expressed as $q_1(t) = \breve{a}_1(t)e^{-i\omega_1 t}$, where $\breve{a}_1(t) = a_1(t)e^{-i\theta}$ is the complex time-dependent amplitude of vibration and $i = \sqrt{-1}$. For a small value of dissipation, $a_1(t)$ varies slowly over the periods of oscillation such that it can be approximated as a constant, i.e., $a_1(t) \sim A_1$. Inserting $q_1(t)$ and its time derivative, $\dot{q}_1(t)$ in Eq. 3, we can write

$$\langle (P_1)^{th}_{\mathbf{flu}}(t)\rangle_{neq} = -\left(k_f - \omega_1 \tilde{c}^I_f(\omega_1)\right)\langle q_1(t)\rangle_{neq} - \tilde{c}_f(\omega_1)\langle \dot{q}_1(t)\rangle_{neq}. \tag{4}$$

Here, $\tilde{c}_f$ and $\tilde{c}^I_f$ are the real and imaginary Fourier transform of $c_f(t)$, respectively, from time to frequency domain and evaluated at ω_1. $\tilde{c}_f$ can be calculated from the slope of $\langle (P_1)^{th}_{\mathbf{flu}}(t)\rangle_{neq}$ with $\langle \dot{q}_1(t)\rangle_{neq}$. The fluid dissipation, $D^{th}_{\mathbf{flu}}$ can be computed using the hydrodynamic force from Eq. 4 and expressed in terms of c_f as

$$D^{th}_{\mathbf{flu}} = \int_{0}^{2\pi/\omega_1} \langle (P_1)^{th}_{\mathbf{flu}}(t)\rangle_{neq}\langle \dot{q}_1(t')\rangle_{neq}\, dt' = \pi\omega_1 A_1^2 \tilde{c}_f(\omega_1). \tag{5}$$

Using Eq. 5 and $E_{sto} = \frac{1}{2}\xi_1 m^0(\omega_1^0)^2 A_1^2$, the inverse quality factor can be calculated as

$$(Q^{-1})^{th}_{\mathbf{flu}} = \frac{1}{2\pi}\frac{D^{th}_{\mathbf{flu}}}{E_{sto}} = \frac{\omega_1 \tilde{c}_f(\omega_1)}{\xi_1 m^0(\omega_1^0)^2}. \tag{6}$$

S.6. FLUID DAMPING KERNEL USING LINEAR RESPONSE THEORY

Linear response theory (LRT)[9,10] states that the response of the thermally equilibrated system to a small external perturbation can be predicted from the thermal fluctuations of the system at equilibrium. In case of the SWCNT resonator with frozen atoms, flexural motion is set by artificially moving the atoms along the mode shape ϕ_1 with a time dependence as $A_1 \sin(\omega_1 t)$. During the nonequilibrium process, the Hamiltonian of the fluid in the coupled system can be written as $\mathcal{H} = \mathcal{H}_0 + \mathcal{A}\lambda(t)$. Here, $\mathcal{H}_0$ is the Hamiltonian before imparting any perturbation. $\lambda(t)$ is the external perturbation and $\mathcal{A}$ is any observable conjugate of λ with respect to the Hamiltonian. In the present case, $\lambda(t) \equiv A_1 \sin(\omega_1 t)$ and $\mathcal{A}$ is the hydrodynamic force by the fluid on the resonator atoms along mode ϕ_1 in response to the perturbation, i.e, $\mathcal{A} \equiv P_1$. Under this setup, LRT can be used to express the response of another physical observable $\mathcal{B}$ in the nonequilibrium state as[9] $\langle \Delta\mathcal{B}(t)\rangle_{neq} = \int_{-\infty}^{\infty} dt'\, \chi_{\mathcal{AB}}(t-t')\lambda(t')$. Here $\langle \Delta\mathcal{B}(t)\rangle_{neq} = \langle \mathcal{B}(t)\rangle_{neq} - \langle \mathcal{B}(t)\rangle_{eq}$, and $\langle . \rangle_{neq}$ and $\langle . \rangle_{eq}$ are nonequilibrium and equilibrium ensemble averages, respectively. $\chi_{\mathcal{AB}}$ is the after-effect function given by $\chi_{\mathcal{AB}}(t) = -\beta \frac{d}{dt}\langle \delta\mathcal{A}(0)\delta\mathcal{B}(t)\rangle_{eq}$, where $\beta = (k_B T)^{-1}$, $\delta\mathcal{A} = \mathcal{A} - \langle \mathcal{A}\rangle_{eq}$ and $\delta\mathcal{B} = \mathcal{B} - \langle \mathcal{B}\rangle_{eq}$ are the thermal fluctuations in variables $\mathcal{A}$ and $\mathcal{B}$ respectively. Presently, we are interested in the response of $P_1(t)$ itself such that $\langle \Delta\mathcal{B}(t)\rangle_{neq} \equiv \langle \Delta P_1(t)\rangle_{neq} = \langle P_1(t)\rangle_{neq} - \langle P_1(t)\rangle_{eq}$ and $\chi_{\mathcal{AB}}(t) \equiv -\beta \frac{d}{dt}\langle \delta P_1(0)\delta P_1(t)\rangle_{eq}$. Noting that $\langle \Delta P_1(t)\rangle_{neq}$ can also be parameterized in terms of any $c_f(t)$ as $\langle \Delta P_1(t)\rangle_{neq} = \int_{-\infty}^{t} dt'\, c_f(t-t')\dot{\lambda}(t')$, it can be shown that

$$c_f(t) = \beta \langle \delta P_1(0)\delta P_1(t)\rangle_{eq}. \tag{7}$$

Similar to Eq. 5, $c_f(t)$ is used to calculate dissipation as a function of frequency, Ω as

$$D = \pi\Omega A_1^2 \int_0^{\infty} dt'\, c_f(t)\cos(\Omega t) = \pi\Omega A_1^2 \tilde{c}_f(\Omega), \tag{8}$$

and consequently, the inverse quality factor, $Q^{-1} = \Omega\tilde{c}_f(\Omega)/(\xi_1 m^0 (\omega_1^0)^2)$ using Eq. 6.

REFERENCES

[1] D. Bolmatov, M. Zhernenkov, D. Zavyalov, S. N. Tkachev, A. Cunsolo, and Y. Q. Cai, Scientific Reports **5** (2015).

[2] Z. Tang, H. Zhao, G. Li, and N. Aluru, Physical Review B **74**, 064110 (2006).

[3] H. Zhao, Z. Tang, G. Li, and N. Aluru, Journal of Applied Physics **99**, 064314 (2006).

[4] M. Born, K. Huang, and M. Lax, American Journal of Physics **23**, 474 (1955).

[5] F. Bloch, Zeitschrift für Physik A Hadrons and Nuclei **52**, 555 (1929).

[6] B. Hauer, C. Doolin, K. Beach, and J. Davis, Annals of Physics **339**, 181 (2013).

[7] Z. Wang, J. Wei, P. Morse, J. G. Dash, O. E. Vilches, and D. H. Cobden, Science **327**, 552 (2010).

[8] M. Tuckerman and B. Berne, The Journal of Chemical Physics **98**, 7301 (1993).

[9] D. Frenkel and B. Smit, *Understanding molecular simulation: from algorithms to applications*, vol. 1 (Academic Press, 2001).

[10] M. Paul and M. Cross, Physical Review Letters **92**, 235501 (2004).
